# Modulating the gut microbiota and inflammation is involved in the effect of diosgenin against diabetic nephropathy in rat

**DOI:** 10.3389/fphar.2025.1555849

**Published:** 2025-05-19

**Authors:** Jiang Shanshan, Pan Shu, Hu Xiao, Kudelaidi Kuerban, Zhu Hao, Wang Yujie, Wang Rong, Shi Yuhuan, Yuan Yongfang

**Affiliations:** ^1^ Department of Pharmacy, Shanghai Ninth People’s Hospital, Shanghai JiaoTong University School of Medicine, Shanghai, China; ^2^ State Key Laboratory of New Drug and Pharmaceutical Process, Shanghai Institute of Pharmaceutical Industry, China State Institute of Pharmaceutical Industry, Shanghai, China; ^3^ School of Life Science and Technologies, Tongji Hospital, Tongji University, Shanghai, China

**Keywords:** diosgenin, anti-inflammatory, gut microbiota, diabetic nephropathy, NLRP3 inflammasome

## Abstract

**Background:**

Diabetic nephropathy (DN) is a severe complication of diabetes, which has been increasingly associated with gut microbiota dysbiosis and inflammatory dysregulation.

**Objective:**

This study investigates the dual therapeutic potential of diosgenin (DIO), a steroidal sapogenin, in modulating the gut-kidney axis and NLRP3 inflammasome activity in a streptozotocin (STZ)-induced DN rat model.

**Methods:**

Oral DIO administration (20 mg/kg, 8 weeks) was used to treat the DN rats. The study assessed the effects on metabolic and renal function parameters, renal apoptosis and fibrosis, gut microbiota diversity, and NLRP3 inflammasome activation in the kidney.

**Results:**

DIO treatment ameliorated the progression of DN, improving metabolic and renal function. It attenuated renal apoptosis and fibrosis and restored gut microbiota diversity, particularly enriching the abundance of *Lachnospiraceae* and *Eubacterium*. Mechanistically, DIO suppressed NLRP3 inflammasome activation in the kidney, disrupted the LPS-TLR4/NF-κB signaling cascade, and reduced systemic pro-inflammatory cytokines (IL-1β, IL-6).

**Conclusion:**

DIO is a multitarget agent that addresses both gut microbiota homeostasis and NLRP3-driven inflammation, presenting a novel therapeutic strategy for DN through modulation of the gut-kidney axis.

## 1 Introduction

Diabetic nephropathy (DN) is a critical complication of both type 1 and type 2 diabetes and the most common cause of end-stage renal disease (ESRD) worldwide. Various factors, including hyperglycemia, genetic predisposition, metabolic abnormalities involving polyols, dietary factors, insulin resistance, and endoplasmic reticulum stress, among others, play roles in DN progression (Sagoo and Gnudi, 2020). There are therapeutic challenges associated with DN due to an unclear relationship between mediators and multiple pathways in its development process (Samsu, 2021). Consequently, there is an urgent need for the exploration of effective therapeutic agents and the elucidation of potential mechanisms to address the management of DN.

Various studies have established that disturbances in metabolic processes and inflammatory responses, typical of both T1DM and T2DM, play significant roles in the development of DN (Rayego-Mateos et al., 2020), where inflammation and immune response play key roles (Rayego-Mateos et al., 2023). Wu et al. found that higher mRNA levels of NLRP3 inflammasome and IL-1β in diabetic patients with macroalbuminuria are linked to serum creatinine and urinary albumin-to-creatinine ratio, suggesting potential biomarkers (Wu et al., 2022). Similarly, renal biopsy tissues from DN patients exhibited upregulation of IL-1β, NLRP3, and interleukin-18 (IL-18) (Wu et al., 2022). Experimental induction of DN in rats through a high-fat diet/streptozotocin regimen showed elevated expression of IL-1β, NLRP3, and caspase 1, accompanied by severe kidney damage (Han et al., 2021; Zhou et al., 2020). Inhibition of NLRP3 expression alleviated glomerular hypertrophy, sclerosis, mesangial dilatation, interstitial fibrosis, and glucose-induced inflammation (Hou et al., 2020). Hence, the NLRP3 inflammasome is a potential target for interventions in DN. In addition, a range of factors, including reactive oxygen species (ROS), advanced glycation end products (AGEs), profibrotic cytokines such as transforming growth factor-beta, inflammatory agents, and the enzyme protein kinase C (PKC), have been identified as playing a role in the advancement of DN (Ma et al., 2022), with notable impact from cytokines including interleukins and interferons (Rayego-Mateos et al., 2020).

Recent studies have pointed out the role of gut microbiota modification and inflammation pathway activation in the development of DN, suggesting a potential relationship between the gut-kidney axis, although the causality needs to be further investigated (Das and Gnanasambandan, 2023). Dysbiosis disrupts gut homeostasis, diminishing villi length, goblet cell count, and cell-tight junction proteins, while promoting gut wall fibrosis. Moreover, studies indicated the involvement of the gut-kidney axis in DN, with *Ruminococcus gnavus* potentially exacerbating diabetic nephropathy by influencing uremic toxin levels and fostering inflammation (Hong et al., 2024). Therefore, early kidney complications linked to diabetes may be mitigated through gut microbiota modulation.

Emerging data has highlighted the health advantages of natural phytochemicals, which have the potential to treat a wide range of disorders, including DN (Kushwaha et al., 2020). DIO, a steroidal sapogenin from the *Dioscorea* plant family, mitigate diabetes risk and modulating pathways involved in inflammation, apoptosis, oxidative stress, and glycolipid metabolism to ameliorate DN (Gan et al., 2020). Studies have demonstrated DIO’s protective effects in the early stages of DN by modulating SIRT6 (Wang et al., 2022). Additionally, dioscin, another compound, has shown protective effects against DN by targeting renal inflammation, oxidative stress, and apoptosis through various pathways (Cai et al., 2020; Zhong et al., 2022). In our previous study, DIO can be used as a microecological regulator to induce antitumor immunity by increasing the abundance of *Clostridiales* order and reducing the abundance of *Bacteroidales* order in C57BL/6 mice (Dong et al., 2018). It can also be found that *Lactobacillus* was obviously upregulated. Tong et al. has found that *Lactobacillus rhamnosus* GG derived extracellular vesicles could ameliorate intestinal inflammation by inhibiting TLR4-NF-κB-NLRP3 axis activation. Our published research invested that inhibition of the NLRP3 inflammasome was confirmed to reduce renal fibrosis in DN rats (Pan et al., 2024). However, prior studies have focused narrowly on DIO’s suppression of NF-κB signaling or taxonomic shifts in gut bacteria, neglecting its potential to target NLRP3-microbiota crosstalk, a novel axis we address here (He et al., 2022). This study is the first to establish that DIO alleviates DN by integrating NLRP3 inflammasome inhibition with gut microbiota remodeling, a dual mechanism distinct from earlier reports. Consequently, we hypothesize that DIO emerges as a potential bioactive agent for DN management by exploiting prebiotic-like effects on intestinal bacteria. Hence, we investigated the protective impact of DIO in preventing DN caused by streptozotocin (STZ) in rat model and underlined the potential mechanism by which DIO impacts DN through changes in the gut flora. The study intended to understand the complicated interaction and mutual effect between intestine and renal health.

## 2 Materials and methods

### 2.1 Materials and reagents

Diosgenin, sourced from Nanjing Spring & Autumn Biotech Co., Ltd. (Jiangsu, China), displayed a purity level greater than 98%, as verified by high-performance liquid chromatography (HPLC). streptozotocin (STZ) and pioglitazone (PGZ) were acquired from Sigma-Aldrich, located in St. Louis, Missouri, United States. Additionally, a full array of primary and secondary antibodies was sourced from Abcam plc, based in Cambridge, United Kingdom. The remaining analytical-grade chemicals used in the study were purchased from well-regarded regional chemical suppliers.

### 2.2 Animals and model establishment

The study’s animal-related methods were sanctioned by the Animal Ethics Committee of Shanghai Ninth People’s Hospital, designated with the approval number SH9H-2022-A047-SB. Male Sprague-Dawley rats, aged between 5 and 6 weeks and with weights ranging from 120 to 150 g, were obtained from the Laboratory Animal Services Division of the Shanghai Institute of Family Planning in China. These animals were maintained under specific pathogen-free (SPF) conditions, where each cage contained either two or a few individuals. The environmental temperature was regulated between 20 and 24°C with a consistent 12-hour alternating light and dark schedule. The animals were provided with unlimited access to nourishment and hydration throughout the duration of the study. Following a week-long acclimation phase, the rats in the test group received a solitary intraperitoneal injection of streptozotocin (STZ), dosed at 75 mg/kg of body mass, dissolved in a 0.1 M citrate buffer at a pH of 4.5. Animals exhibiting a fasting blood glucose level exceeding 16.7 mmol/L in the tail vein, as measured by the glucometer OneTouch Ultra from Johnson & Johnson (United States), were classified as having diabetes. The rodents were then randomly allocated to one of four therapeutic regimens (*n* = 6) for an 8-week experimental period. Thereafter, the diabetic animals were weighed and evenly apportioned across four study groups: a standard control group, a group with DN, a cohort receiving DIO (administered at 20 mg/kg daily), and a cohort receiving pioglitazone (PGZ, also administered at 20 mg/kg daily). Throughout the study’s timeline, all rodents adhered to their designated dietary plans. Animals in the DIO and PGZ groups were administered diosgenin or pioglitazone through gastric intubation for a period of 8 weeks, whereas those in the control and DN groups were given an equivalent measure of physiological saline (0.9%). At the end of study, the rats were euthanized by an intraperitoneal injection of 10% chloral hydrate under anesthesia.

Throughout the study, both the body mass and fasting blood glucose concentrations were tracked on a weekly basis. Weight was documented fortnightly, with periodic checks of glucose levels in the blood during fasting occurring on a bi-weekly schedule. Following the conclusion of the research, samples of urine and feces, collected throughout a full day, were refrigerated at −80°C. Blood samples of rats were collected from tail vein after overnight fasting and centrifuged at 1,500 *g* for 15 min to obtain serum and stored at −80°C. Furthermore, extracted tissues and organs were submerged in a formaldehyde solution and similarly kept at the exceedingly low temperature of −80°C for future examination.

### 2.3 Biochemical assays of serum and urine sample

Determination of glucose and creatinine levels in serum was accomplished using diagnostic kits sourced from commercial kits, adhering to their standard procedures. Quantification of urinary microalbumin and urea nitrogen was performed using commercial kits, following the manufacturer’s guidelines.

### 2.4 Tissue collection and histopathology

Kidney specimens were sectioned longitudinally and then submerged in a 4% paraformaldehyde solution for overnight fixation. This was followed by encapsulation in paraffin, from which sections of 4 μm in thickness were extracted. The examination of structural changes was carried out with Hematoxylin-Eosin (H&E), Periodic Acid-Schiff (PAS), and Masson’s Trichrome staining techniques.

### 2.5 TUNEL assay

Cell death was evaluated through the TUNEL method, employing a detection kit from Beyotime Biotechnology. Sections that were fixed in paraffin were first cleared of wax, underwent rehydration, and were treated with proteinase K for a duration of 30 min at a temperature of 37°C. Subsequently, the samples were made permeable with a solution of 0.5% Triton X-100 for a period of 15 min. The cell nuclei were then marked with DAPI and examined under an Olympus fluorescence microscope, originating from Tokyo, Japan.

### 2.6 Immunohistochemistry analysis

Kidney tissue specimens were preserved in a solution of 10% formaldehyde that was neutralized and then encased in paraffin for cutting into sections with a thickness of 5 μm, in anticipation of immunohistochemical examination. The sections were cleared of wax with xylene and ethanol, followed by three washes in phosphate-buffered saline (PBS). Post-blocking with 10% fetal bovine serum at 37°C for half an hour, the sections were treated with the primary antibodies-fibronectin, Collagen IV, IL-1β, TNF-α, and IL-6-at respective dilutions (1:500 for fibronectin, Collagen IV, and IL-6; 1:200 for TNF-α; 1:500 for IL-1β) throughout the chill of the night at 4°C. Following thorough cleansing with PBS on numerous occasions, the sections of tissue were processed with secondary antibodies linked to HRP for a period of 30 min. The DAB reagent was applied to reveal regions of positive immunostaining. Visual records were then secured using an Olympus IX73 microscope, paired with a DP80 camera, both manufactured by Olympus Corp., Tokyo, Japan.

### 2.7 Western blot (WB) analysis

Total kidney protein was isolated and quantified with the BCA Protein Assay Kit. The protein samples were then applied to polyvinylidene fluoride membranes. After a 2-hour incubation period with 5% skim milk in Tris-buffered saline with Tween (TBST), the membranes were incubated with secondary antibodies for an additional 2 h. Protein detection was accomplished via a WB protocol, with band intensities normalized against an internal control.

### 2.8 Caecal microbiota analysis

Genomic DNA from bacteria was isolated utilizing the Fast DNA SPIN Kit by MP Biomedicals (Santa Ana, CA, United States) following the guidelines provided by the manufacturer, followed by preservation at −20°C for subsequent analysis.

The amplification of the V3-V4 segment of the bacterial 16S rRNA gene was executed with primers 338F (5′-ACT​CCT​ACG​GGA​GGC​AGC​A-3′) and 806R (5′-GGACTACHVGGGTWTCTAAT-3′), each with a unique 7-bp barcode for multiplexed sequencing. The cleanup of PCR outcomes was conducted with Agencourt AMPure Beads by Beckman Coulter (Indianapolis, IN), while the measurement of concentration was accomplished using the PicoGreen dsDNA Assay Kit from Invitrogen (Carlsbad, CA, United States). After determining the quantity of each sample, the amplified DNA fragments were mixed in equal measures for sequencing on the Illumina MiSeq system with a 2 × 300 bp setup, employing the MiSeq Reagent Kit v3, carried out by Shanghai Personal Biotechnology Co., Ltd. in Shanghai, China. The resulting sequences were analyzed employing the QIIME workflow (version 1.8.0), as detailed earlier (Hong et al., 2024).

### 2.9 Statistical analysis

Outcomes derived from no fewer than three separate trials are displayed as average ± standard deviation. Analytical assessments were performed utilizing Student’s t-test or one-way ANOVA through GraphPad Prism version 6.0 (GraphPad Software Inc., San Diego, CA, United States). A threshold for statistical significance was established at a *p*-value below 0.05.

## 3 Results

### 3.1 DIO administration ameliorated glucose metabolism and kidney injury in DN rat

**FIGURE 1:**
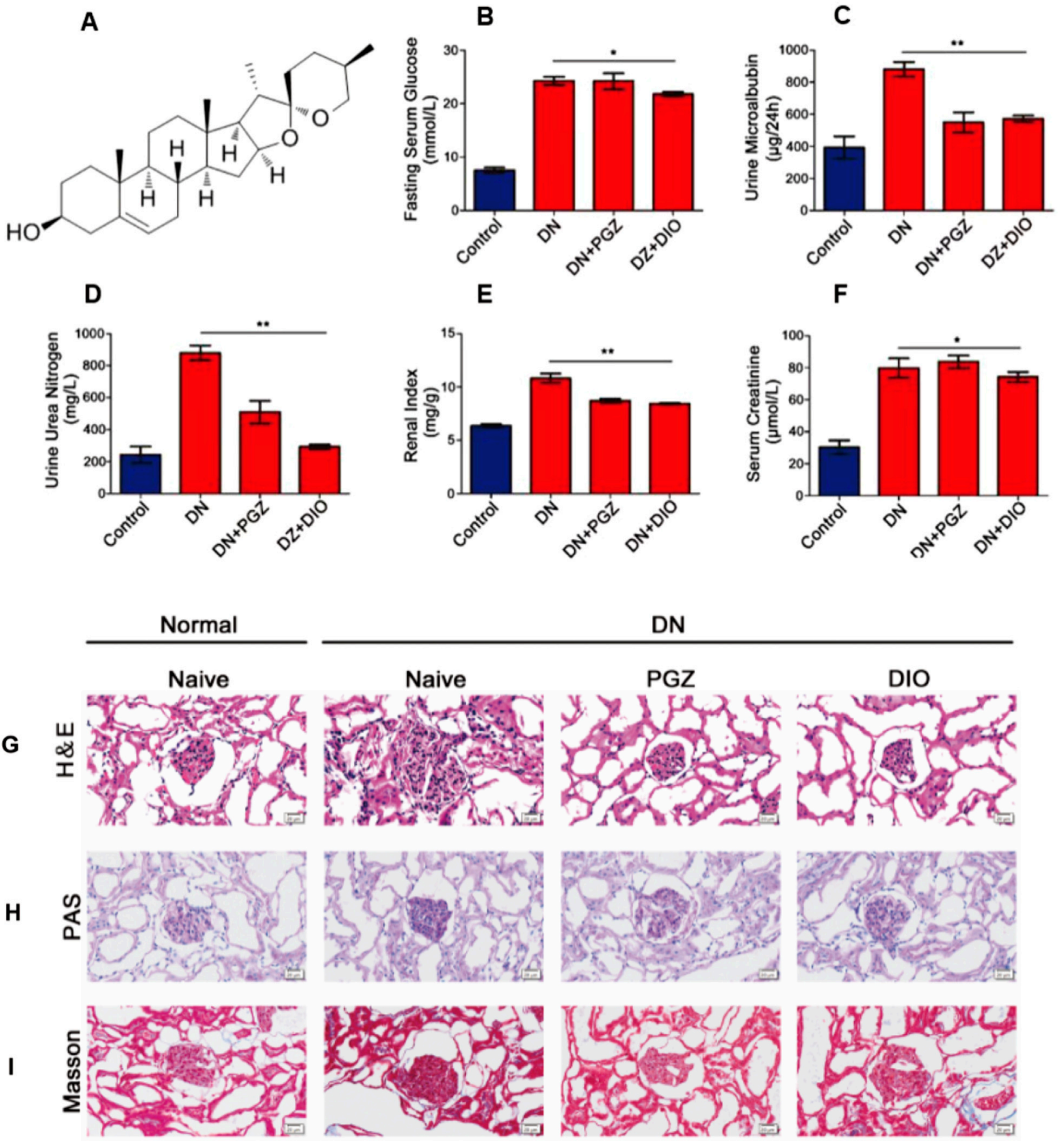
DIO ameliorated glucose metabolism and kidney injury in DN rats after 8 weeks of administration. **(A)** The structure of diosgenin (DIO). **(B)** Serum glucose, **(C)** Urine microalbumin. **(D)** Serum creatinine. **(E)** Urine urea nitrogen, **(F)** The renal index. **(G)** H&E staining, **(H)** PAS staining and **(I)** Masson staining of representative kidney sections, photographed at ×200 magnification. ^*^ *P* < 0.05, ^**^ *P* < 0.01, ^***^ *P* < 0.001, compared with DN group.

Fasting serum glucose (FBG) serves as a hallmark of diabetes. We utilized FBG to gauge the impact of DIO on abnormal glucose metabolism. Our findings indicated a marked decrease in fasting blood glucose levels in rats with diabetic nephropathy that received DIO, as opposed to those in the diabetic nephropathy group alone (*P* < 0.05) (Figure 1B). For the assessment of kidney injury and functionality, we quantified urinary microalbumin, serum creatinine, and urinary urea nitrogen (Figures 1C–E). Within the diabetic nephropathy cohort, these indicators were notably higher than in the control cohort. Conversely, the administration of diosgenin (DIO) resulted in a decrease in these biomarker levels.

In the cohort that received pioglitazone (PGZ), there was a noticeable reduction in the levels of microalbumin in urine and urea nitrogen compared to those with diabetic nephropathy (DN). However, the levels of creatinine in serum were not altered by the PGZ regimen. Additionally, the renal index was observed to be markedly increased in the DN group in contrast to the control group (*P* < 0.01), and it was significantly diminished in the group administered diosgenin (DIO) versus the DN group (*P* < 0.01) (Figure 1F).

### 3.2 DIO reduced renal histopathological lesions in DN rat

Histological analysis using H&E staining demonstrated typical DN pathological features in the model group, encompassing conditions like enlargement of the glomeruli, cell multiplication, buildup of the extracellular matrix, and thickening of the basement membrane. Nevertheless, the addition of PGZ and DIO mitigated these manifestations (as shown in Figure 1G). Periodic Acid-Schiff (PAS) staining exposed substantial sugar accumulation and excessive mesangial expansion (Figure 1H). Additionally, Masson’s trichrome staining indicated renal fibrosis, which improved following treatment (Figure 1I).

### 3.3 DIO blocked STZ-induced cell apoptosis of kidney in STZ-induced DN rat

**FIGURE 2:**
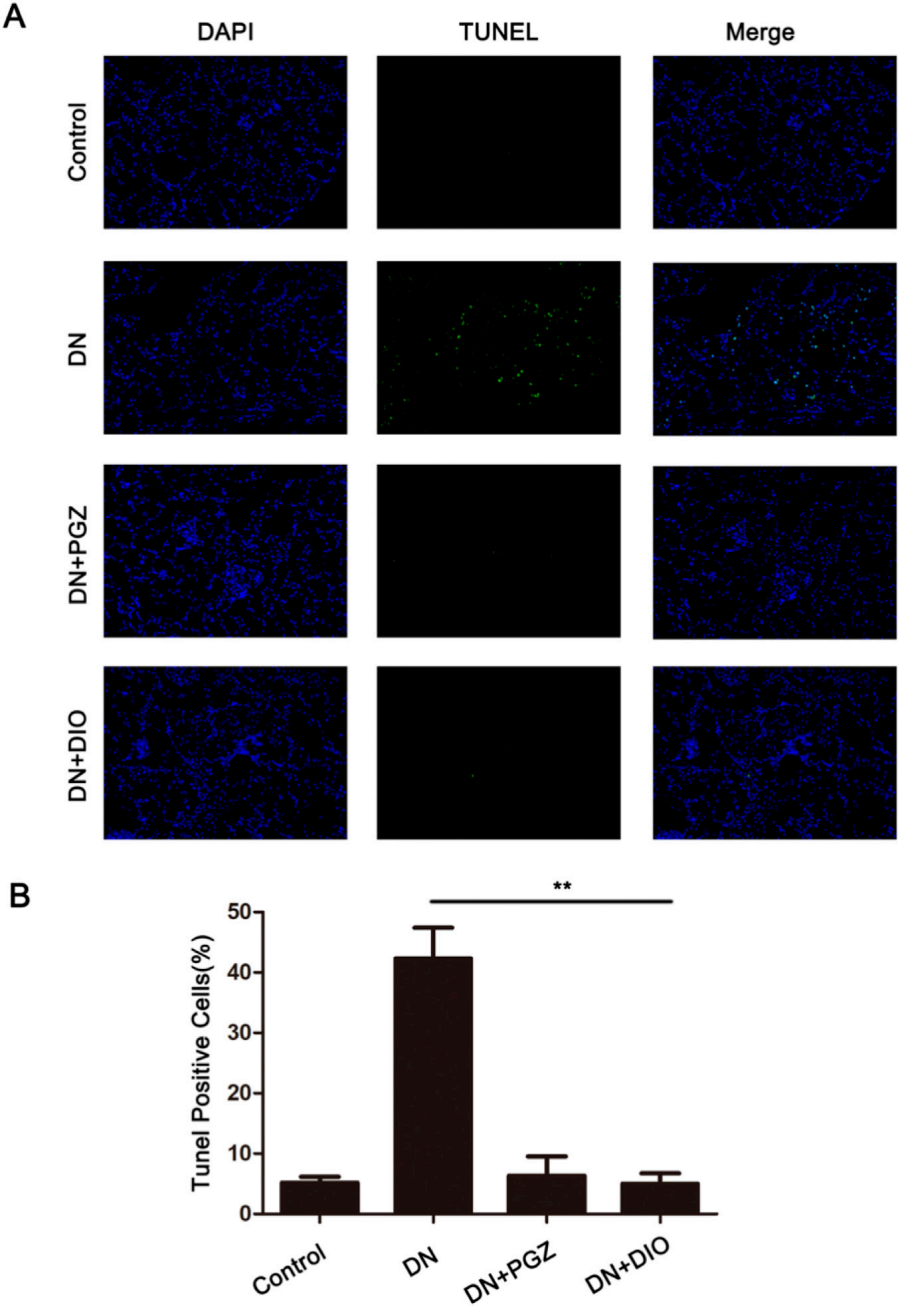
DIO blocked STZ-induced cell apoptosis of kidney in DN rats. **(A)** The kidney sections were stained with TUNEL and measured by confocal microscope. The apoptotic cells showed green fluorescence (200×). **(B)** The statistics of TUNEL-positive cells (%). ^*^ *P* < 0.05, ^**^ *P* < 0.01, ^***^ *P* < 0.001, compared with DN group.

To explore whether DIO’s protective effects on mesangial cells involve apoptosis inhibition, we analyzed kidney tissue by using the TUNEL assay. This assay labels apoptotic cells with green fluorescence, indicating DNA strand breaks. In our study, we observed a 37% increase in intense TUNEL-specific fluorescence in the DN group (Figure 2). However, the DIO and PGZ treated groups exhibited minimal TUNEL-specific green fluorescence (*P* < 0.01). These findings suggested that DIO effectively prevented STZ-induced kidney cell apoptosis in DN rats, as evidenced by the TUNEL assay.

### 3.4 DIO reduced the renal fibrosis-associated proteins in DN rat

**FIGURE 3:**
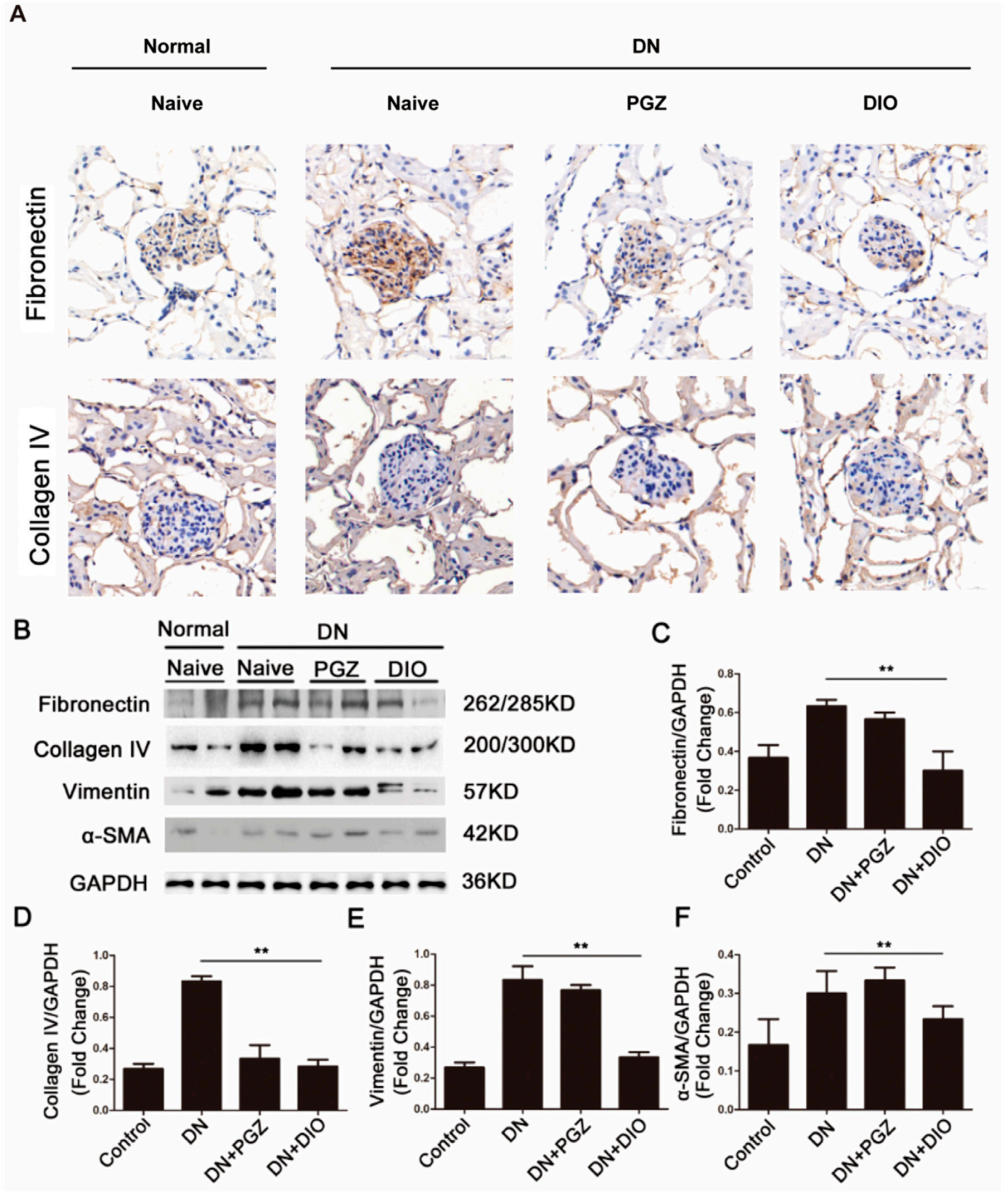
DIO attenuated renal fibrosis in DN. **(A)** Representative immunohistochemical staining of fibronectin and collagen IV in renal tissues in mouse model. **(B)** Renal expression of fibrosis markers including fibronectin, collagenin IV, vimentin, and α-SMA in DN rats. Densitometric analysis of **(C)** fibronectin, **(D)** collagenin IV, **(E)** vimentin, and **(F)** α-SMA. ^*^ *P* < 0.05, ^**^ *P* < 0.01, ^***^ *P* < 0.001, compared with DN group.

Illustrated in Figure 3A, the concentrations of fibronectin and collagen IV, essential constituents of the kidney’s extracellular matrix, were elevated after STZ administration but were attenuated with the addition of PGZ and DIO. Moreover, STZ infusion led to heightened levels of fibrosis indicators in the kidney, such as fibronectin, collagen IV, vimentin, and α-SMA. Conversely, the application of DIO lessened the excessive expression of these indicators in renal tissues, signifying its capacity to ameliorate fibrosis in rats with STZ-provoked diabetic nephropathy. Collectively, these results imply that DIO may possess the ability to reduce renal damage and fibrotic changes in nephropathy triggered by STZ.

### 3.5 DIO downregulated the expression of NLRP3 inflammasome in DN rat

**FIGURE 4:**
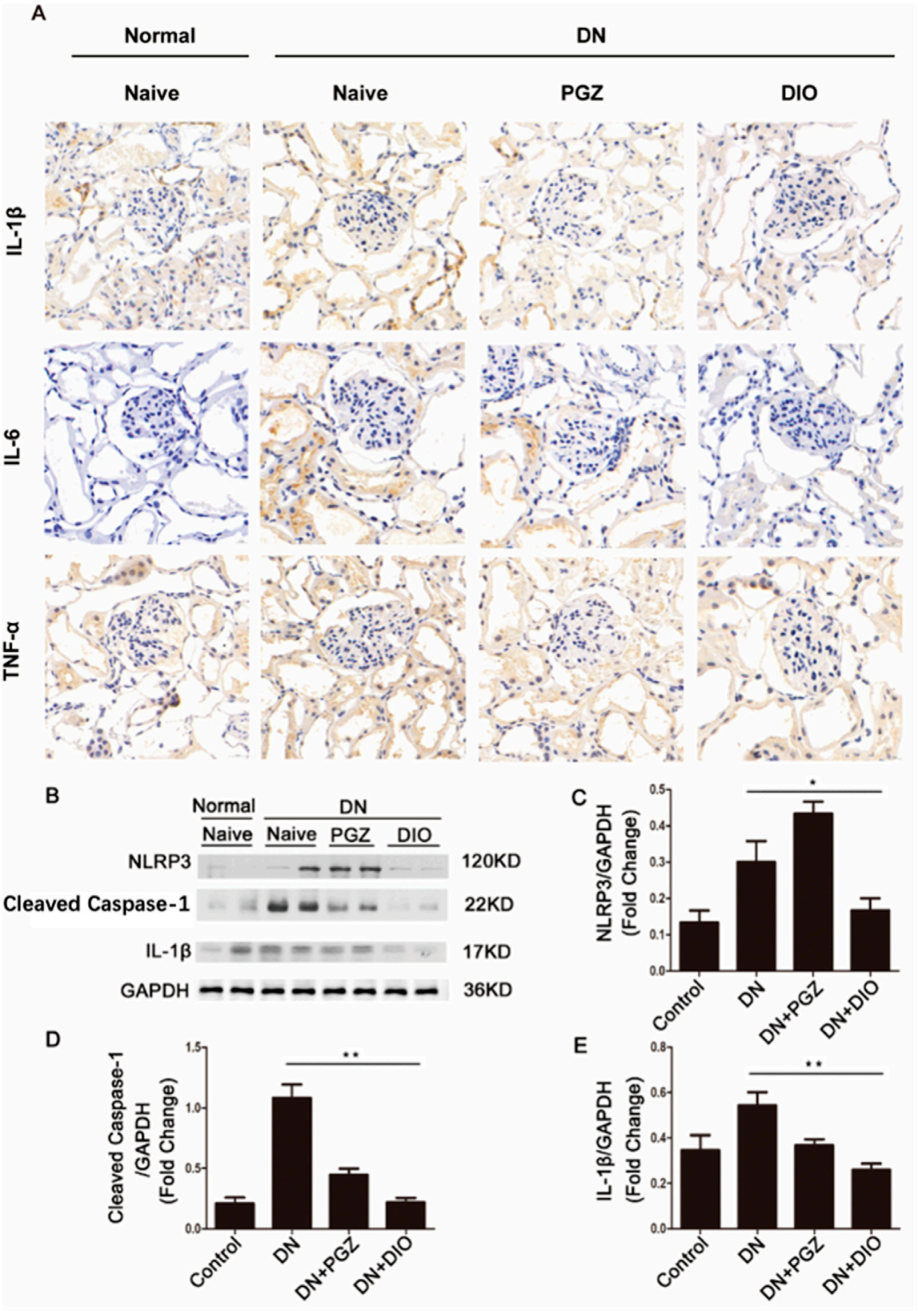
DIO suppressed renal inflammation. **(A)** Immunohistochemical staining of IL-1β, IL-6 and TNF-α in renal tissues. **(B)** Western blot analysis of NLRP3, Cleaved caspase-1 and IL-1β. The ratio of Western blot analysis of **(C)** NLRP3, **(D)** Cleaved caspase-1, **(E)** IL-1β. ^*^ *P* < 0.05, ^**^ *P* < 0.01, ^***^ *P* < 0.001, compared with DN group.

We also explored if DIO could ameliorate diabetic nephropathy (DN) in rats by suppressing the NLRP3 inflammasome in the kidneys. Figure 4A demonstrates that immunohistochemical examination revealed a significant decrease in the levels of IL-1β, IL-6, and TNF-α following DIO administration. Furthermore, immunoblotting indicated that STZ induced a robust response in the renal NLRP3 inflammasome pathway, leading to the processing of caspase-1 and the maturation of IL-1β. Conversely, DIO intervention markedly diminished the heightened expression of NLRP3 inflammasome elements in diabetic nephropathy, suggesting its robust anti-inflammatory effect by curbing NLRP3 inflammasome activation in living organisms. Hence, our research suggested that the activation of the NLRP3 inflammasome is implicated in the onset and advancement of nephropathy induced by STZ. DIO administration demonstrated protective effects on the kidneys by inhibiting this activation.

### 3.6 DIO suppressed the colonic inflammation and improved barrier function in DN rat

**FIGURE 5:**
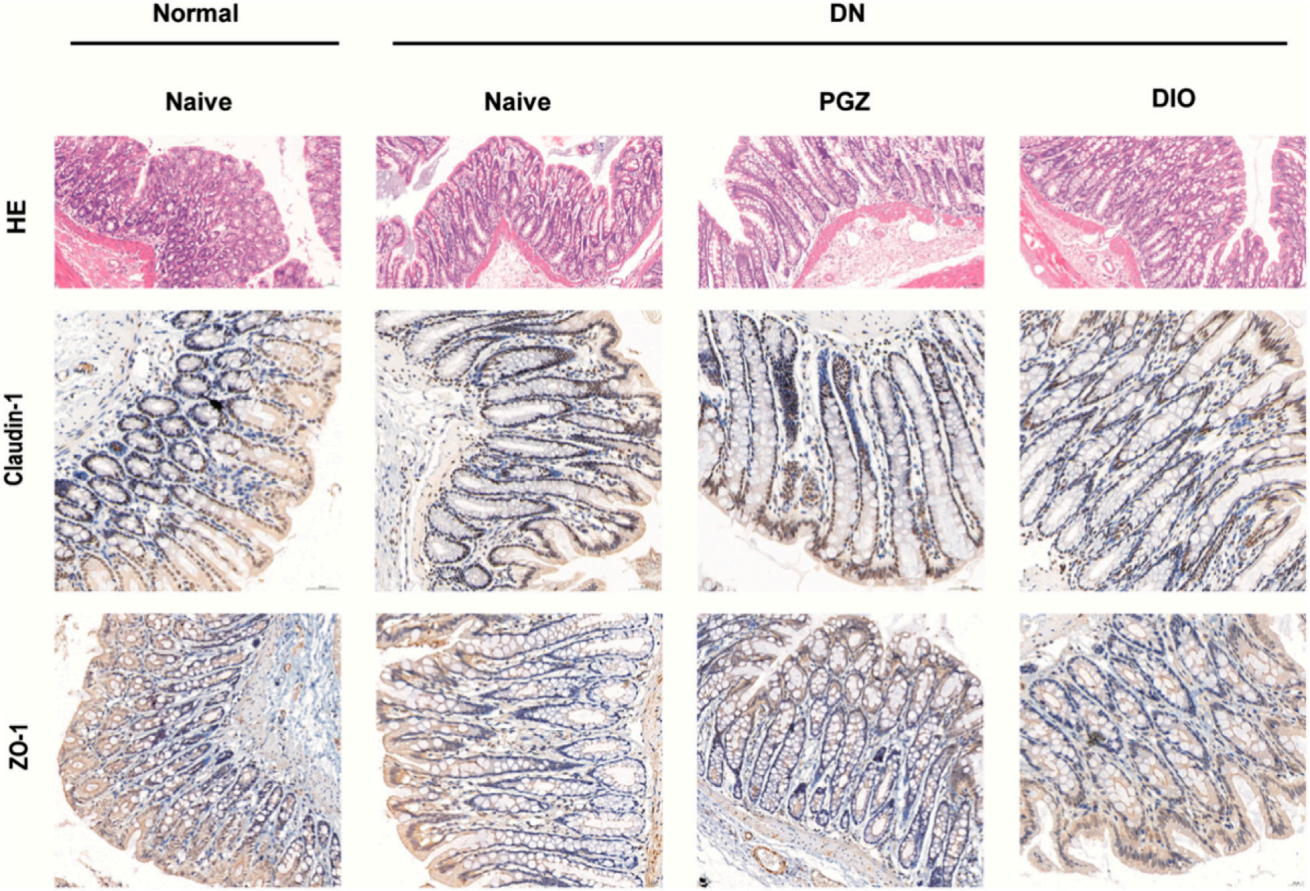
DIO suppressed colon inflammation and improved gut barrier. Representative photomicrographs of HE-stained small intestine sections and photomicrographs of immunofluorescence for Claudlin-1 and ZO-1 at ×200 magnification.

To confirm DIO’s role in mitigating STZ-induced chronic inflammation, we assessed intestinal inflammation and barrier function in DN rats. H&E staining was employed to assess intestinal tissue pathology. Within the colons of STZ-diabetic rat, there was a noted presence of irregular villi and increased goblet cell counts, effects that were mitigated by DIO administration (refer to Figure 5). Furthermore, the impairment of intestinal barrier function in diabetic nephropathy (DN) rodents was validated via staining for occluding junctional proteins claudin-1 and ZO-1. A decrease in the levels of these occluding junctional proteins, claudin-1 and ZO-1, was observed in DN rodents when contrasted with healthy controls. Notably, DIO treatment significantly restored the expression of these proteins in the DN rats (Figure 5). These findings suggest that DIO may protect against gut barrier dysfunction, mucosal inflammation, and endotoxemia.

### 3.7 DIO treatment altered the gut microbiota in DN rat

**FIGURE 6:**
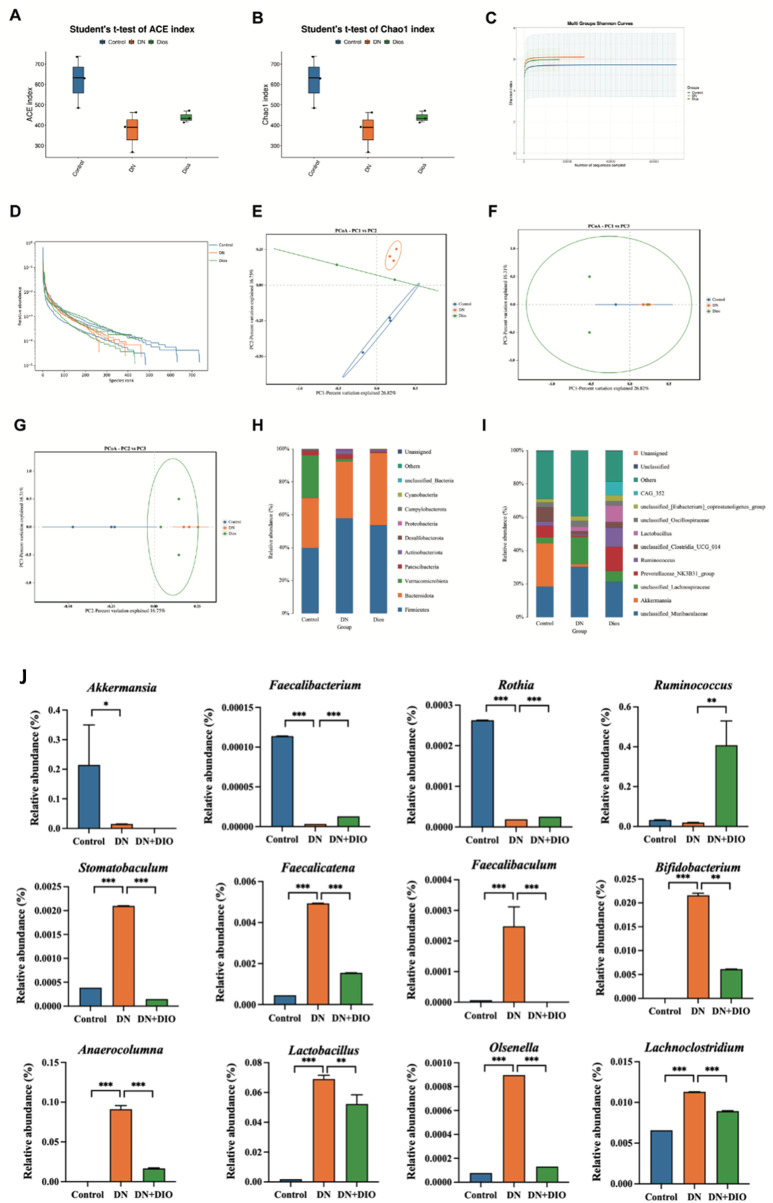
DIO altered gut microbiota in DN rats. **(A)** ACE index. **(B)** Chao1 index. **(C)** Shannon index curve. **(D)** Rank abundance curve. **(E–G)** Weighted UniFrac PCoA (Principal coordinates analysis). **(H)** Relative abundance at phylum level. **(I)** Relative abundance at genus level. **(J)** Relative abundance of the significantly-altered bacteria at the genus level. Data are represented as means ± SD (*n* = 5). ^*^ *P* < 0.05, ^**^ *P* < 0.01 and ^***^ *P* < 0.001 indicated significant difference between compared groups.

Diversity metrics, encompassing rarefaction and rank-abundance graphs, gauged the sufficiency of sequence depth to represent the variety within microbial communities. A coverage rate surpassing 99% across all interventions confirmed the sufficiency of sequence data for comprehensive analysis. DIO rats exhibited significantly higher ACE index and improved Chao index versus DN rats (Figures 6A,B), indicating enhanced rectal bacterial richness and diversity. Most samples reached a plateau, consistent with the rank abundance curve (Figures 6C,D) Following DIO administration, the Chao1 and ACE indices rose compared to the control group. The beta diversity assessment, conducted through Principal Coordinate Analysis (PCoA), highlighted differences between the diabetic nephropathy cohort and the other groups. The introduction of DIO restructured the microbial community composition in rats to resemble that of healthy rats (Figures 6E–G).

To further elucidate the relationships and distinctions among each group, we conducted taxonomy and LEfSe analyses. Figures 6H,I illustrates the bacterial community composition in rectal contents at both the phylum and genus levels. Bacteroidetes and Firmicutes constituted the primary bacterial groups in every cohort. Rats treated with STZ witnessed an upsurge in the Firmicutes to Bacteroidetes ratio. In the diabetic nephropathy cohort, there was a notable decrease in Bacteroidetes and a corresponding increase DN Proteobacteria; however, DIO intervention successfully reversed these trends, bringing the levels closer to those of the control group. A thorough examination at the genus level disclosed a reduced presence of the advantageous microorganism Bifidobacterium among STZ-injected rats. In addition, the DN cluster displayed an escalation in the proportion of *Mucispirillum* and a reduction in *Prevotellaceae*. *Lacobacillus*, viewed as a prebiotic, was more prevalent in both the DIO and diabetic nephropathy clusters. Moreover, in the level of genus, the addition of DIO led to a heightened presence of *Ruminococcus*, *Faecalibacterium* and *Rothia*, (Figure 6J). DIO selectively depletes taxa implicated in DN progression, including those driving inflammation (e.g., *Lachnoclostridium*), gut barrier disruption (e.g., *Faecalibaculum*), and uremic toxin synthesis (e.g., *Stomatobaculum*) (Figure 6J).

## 4 Discussion

Characterized by an ongoing deterioration in renal capabilities, diabetic nephropathy is marked by ongoing leakage of small amounts of albumin in the urine, a decline in the Glomerular Filtration Rate, and a heightened ratio of urinary albumin to creatinine levels (Thipsawat, 2021). Diosgenin, a steroidal sapogenin with significant pharmacological potential, is predominantly extracted from plants of the *Dioscorea genus* (yam family), such as *Dioscorea zingiberensis* (Chinese wild yam) and *Dioscorea nipponica* (Japanese yam). These species are considered the richest natural sources, with diosgenin content ranging from 1% to 8% in their rhizomes depending on the plant species, growth conditions, and extraction methods (Tang et al., 2013; Yi et al., 2014). Additionally, diosgenin is found in lower quantities in other plants, including fenugreek (*Trigonella foenum-graecum*) and certain Solanum species (e.g., *Solanum incanum*), where it is often bound to sugar moieties as saponins (Ou-Yang et al., 2018). Preclinical studies have highlighted its promising anti-tumor, anti-inflammatory, and antioxidant effects, demonstrating efficacy in managing cardiovascular disease, type 2 diabetes, and neurodegenerative disorders (Semwal et al., 2022). Experimental results revealed that STZ-induced rats exhibited increased blood glucose levels, kidney index, serum creatinine, and urine albumin, mirroring DN characteristics, all of which were significantly reduced with DIO supplementation. Additionally, various studies indicated upregulation of inflammation markers (Higo et al., 2008). Notably, DIO administration significantly ameliorated the histopathological changes induced by DN.

Research has shown that DN alters apoptotic markers, encompassing the heightened expression of Bcl2-linked cell death promoter and Bcl-2-linked X protein, along with increased mitochondrial membrane permeability (MMP), elevation of caspase-3 and -9, and other factors (Ma et al., 2022). TUNEL results revealed a higher number of apoptotic cells (green fluorescence) in DN rats, which was reduced with DIO treatment. DN progression involves various pathological mechanisms such as oxidative stress, inflammatory cell infiltration, inflammation, and fibrosis. Among these, targeting fibrosis has proven effective in DN prevention (Calle and Hotter, 2020). Rats with DN displayed increased kidney concentrations of fibrotic markers such as fibronectin, collagen IV, vimentin, and α-SMA, levels that were attenuated by DIO treatment. Our findings demonstrated that DIO also modulated renal fibrosis parameters, alleviating diabetic nephropathy-induced structural damage in the renal cortex.

Emerging research underscores the dual role of the NLRP3 inflammasome in driving both inflammation and renal fibrosis, pivotal mechanisms in DN progression (Zhang and Wang, 2019). Our study demonstrated that inhibition of NLRP3 inflammasome activity by DIO alleviated renal inflammation and scarring in DN rats, evidenced by suppressed caspase-1 activation, reduced IL-1β levels, and diminished expression of fibrotic markers (fibronectin and collagen IV). These findings suggested that DIO attenuates extracellular matrix (ECM) accumulation and mesangial expansion by targeting the NLRP3 pathway. Importantly, systemic and local inflammation, recognized as central drivers of DN (Xue et al., 2023), may further exacerbate renal damage through gut-kidney crosstalk. In DN rats, gut barrier dysfunction-characterized by intestinal villi atrophy and increased permeability-likely facilitates bacterial translocation and leakage of pathogen-associated molecular patterns (e.g., LPS) into circulation, amplifying systemic inflammation via TLR4/NF-κB signaling. Consistent with this, we observed elevated renal TNF-α, IL-6, and IL-1β levels in STZ-treated rats, alongside NLRP3 pathway activation. DIO administration not only mitigated renal inflammation but also restored gut barrier integrity, enhancing mucosal repair and capillary density in the small intestine. This dual protection aligns with reports that DIO suppresses the TLR4-MyD88-NF-κB axis (Jin et al., 2022), thereby reducing endotoxemia and interrupting the vicious cycle of gut-derived inflammation and renal injury. Thus, DIO’s therapeutic efficacy in DN may arise from its concurrent targeting of NLRP3-driven renal fibrosis and gut barrier preservation, highlighting the interconnectedness of mucosal immunity and chronic kidney disease pathogenesis.

The gut microbiota is crucial for the complex interaction between the gut and metabolic systems (Schoeler and Caesar, 2019). Imbalances in the microbial community are commonly detected in a range of autoimmune and metabolic conditions, including bowel inflammation, persistent kidney ailments, high blood sugar levels, excess body weight, and heart-related illnesses (Li et al., 2017). The gut microbiota alterations observed in our study align with and extend prior findings on dysbiosis in DN. The enrichment of *Proteobacteria* and *Actinobacteria* in the DN group corroborates reports linking these phyla to endotoxin production (e.g., LPS from *Proteobacteria*) and chronic inflammation in renal pathologies (Sun et al., 2021). Notably, LPS from *Proteobacteria* not only disrupts gut barrier integrity but also amplifies systemic inflammation via TLR4/NF-κB signaling, a pathway implicated in renal fibrosis and podocyte injury (Bian et al., 2025). Similarly, the elevated *Actinobacteria* in DN may reflect an imbalance in bile acid metabolism, as certain *Actinobacteria* members (e.g., *Bifidobacterium*) are critical for converting primary bile acids into pro-inflammatory secondary forms (Han et al., 2022). DIO’s ability to suppress these taxa suggested a dual role: mitigating endotoxemia and restoring bile acid homeostasis, which could collectively attenuate renal inflammation.

The DIO-induced increase in *Lachnospiraceae* (e.g., *Ruminococcus* and *Oscillospira*) and butyrate-producing *Eubacterium* highlighted a potential mechanism linking gut microbiota modulation to renal protection (Hong et al., 2024). *Lachnospiraceae* are key producers of short-chain fatty acids (SCFAs), particularly butyrate, which enhances gut barrier function by upregulating tight junction proteins (e.g., occludin) and suppressing NF-κB-driven inflammation (Lyu et al., 2017). Butyrate from *Eubacterium* may further protect against DN by inhibiting histone deacetylases (HDACs) in renal tubular cells, thereby reducing oxidative stress and fibrosis (Han et al., 2022). The rise in *Oscillospira*, a genus inversely associated with obesity and inflammation (Konikoff and Gophna, 2016), aligns with DIO’s reported anti-hyperglycemic effects, possibly mediated through improved insulin sensitivity and reduced adipose tissue inflammation (Gan et al., 2020).

Importantly, the enrichment of *Lactobacillus* and *Prevotella* in the DIO group mirrors findings from studies on prebiotics and anti-diabetic phytochemicals (Han et al., 2023). *Lactobacillus* species are known to suppress pathogenic bacteria (e.g., *Enterobacteriaceae*) via competitive exclusion and bacteriocin production, while *Prevotella* enhances mucin synthesis, reinforcing the gut barrier against LPS translocation (Woting and Blaut, 2016). These shifts likely synergize with DIO’s direct anti-inflammatory properties, such as inhibition of NLRP3 inflammasome activation and downregulation of pro-inflammatory cytokines (e.g., TNF-α, IL-6), to disrupt the “gut-kidney axis” of inflammation in DN. In the differential abundance analysis of Gut Microbiota of DIO, we found that DIO restores gut microbiota homeostasis by enriching SCFA-producing symbionts and depleting inflammation- and toxin-associated taxa, providing a microbial basis for its renoprotective effects in DN (Kimura et al., 2022). These findings align with its observed suppression of renal NLRP3 inflammasome activation and systemic inflammation.

Alpha-diversity indices are essential for assessing and comparing biodiversity, including metrics such as observed OTUs, Chao1, ACE, Simpson, and Shannon indices (Hong et al., 2022). In our investigation, we assessed alterations in related bacteria, noting reductions in Chao1, ACE indices, and Shannon indices in DN rats, indicating decreased alpha-diversity, which significantly increased upon DIO supplementation. Additionally, the heightened *Firmicutes*/*Bacteroidetes* ratio, a widely recognized marker of gut microbiota balance, notably increased in the DN group (Lyu et al., 2017) but was reversed with DIO supplementation.

Moreover, new findings indicate that particular gut bacteria may affect immune-regulating cells in the intestines (IRCs), located within the gut lining and serving as connectors between the gut’s bacterial community and disease mechanisms (Li et al., 2018). In addition to the role of gut microbiota, DIO has been shown to effectively alleviate oxidative stress in DN by regulating NOX4 expression and mitochondrial respiratory chain complex to reduce ROS production. It also inhibits mitochondrial and ER stress-induced apoptosis by restoring mitochondrial membrane potential and down-regulating key apoptotic proteins, providing a potential scheme for the treatment of DN (Zhong et al., 2023). Accordingly, we propose that adding DIO to the diet could potentially decrease bacteria that generate LPS, repair the intestinal barrier, and boost the production of advantageous compounds within metabolic and inflammatory processes by adjusting the gut microbiome. Nonetheless, additional studies are essential to gain more insights into the interplay between renal function and the gut ecosystem.

## 5 Conclusion

This study establishes DIO as a multifaceted therapeutic agent for DN, targeting both gut-kidney axis dysregulation and NLRP3 inflammasome-driven inflammation. In STZ-induced DN rats, DIO administration (20 mg/kg, 8 weeks) significantly reduced hyperglycemia, serum creatinine and urinary albumin, concurrently attenuating renal histopathological lesions. In addition, DIO restored gut microbiota diversity, specifically enriching *Lachnospiraceae* and *Eubacterium*. This correlated with suppressed renal NLRP3 activation, and reduced systemic endotoxemia, thereby inhibiting TLR4/NF-κB signaling. Crucially, DIO’s dual action-gut microbiota modulation and inflammasome inactivation-synergistically disrupted the gut-kidney inflammatory cascade. These findings reposition DIO beyond glycemic control, highlighting its potential as a gut microbiota-stabilizing, multitarget therapy for DN. The concordance between microbiota restoration and renal protection underscores its clinical promise for diabetic complications.

### 5.1 Potential limitation of the study

(1) The study uses a murine model of STZ-induced diabetes, which may not fully represent human DN due to differences in glucose metabolism, immune responses, and gut microbiota between rat and humans.
(2) While the study found correlations between gut microbiota changes and inflammation, the causal relationships between diosgenin, specific bacteria, and renal outcomes are speculative and require further investigation using techniques like metagenomic sequencing and fecal microbiota transplantation (FMT).

## Data Availability

The original contributions presented in the study are included in the article/Supplementary Material, further inquiries can be directed to the corresponding author.
